# Multi-Phase Joint-Angle Trajectory Generation Inspired by Dog Motion for Control of Quadruped Robot

**DOI:** 10.3390/s21196366

**Published:** 2021-09-24

**Authors:** Jungsu Choi

**Affiliations:** Department of Robotics Engineering, Yeungnam University, Gyeongsan 38541, Korea; jschoi@yu.ac.kr

**Keywords:** trajectory generation, multiple gait phase transition, fuzzy logic

## Abstract

Quadruped robots are receiving great attention as a new means of transportation for various purposes, such as military, welfare, and rehabilitation systems. The use of four legs enables a robustly stable gait; compared to the humanoid robots, the quadruped robots are particularly advantageous in improving the locomotion speed, the maximum payload, and the robustness toward disturbances. However, the more demanding conditions robots are exposed to, the more challenging the trajectory generation of robotic legs becomes. Although various trajectory generation methods (e.x., central pattern generator, finite states machine) have been developed for this purpose, these methods have limited degrees of freedom with respect to the gait transition. The conventional methods do not consider the transition of the gait phase (i.e., walk, amble, trot, canter, and gallop) or use a pre-determined fixed gait phase. Additionally, some research teams have developed locomotion algorithms that take into account the transition of the gait phase. Still, the transition of the gait phase is limited (mostly from walking to trot), and the transition according to gait speed is not considered. In this paper, a multi-phase joint-angle trajectory generation algorithm is proposed for the quadruped robot. The joint-angles of an animal are expressed as a cyclic basis function, and an input to the basis function is manipulated to realize the joint-angle trajectories in multiple gait phases as desired. To control the desired input of a cyclic basis function, a synchronization function is formulated, by which the motions of legs are designed to have proper ground contact sequences with each other. In the gait of animals, each gait phase is optimal for a certain speed, and thus transition of the gait phases is necessary for effective increase or decrease in the locomotion speed. The classification of the gait phases, however, is discrete, and thus the resultant joint-angle trajectories may be discontinuous due to the transition. For the smooth and continuous transition of gait phases, fuzzy logic is utilized in the proposed algorithm. The proposed methods are verified by simulation studies.

## 1. Introduction

As robotic technologies, including manufacturing, control, and system integration technologies, have been improved dramatically, many sophisticated and intelligent robotic systems have been developed in recent years. One example is a quadruped robot; the quadruped robots are regarded as a new-trend among many robotics researchers [1,2]. Due to the large base of support and high degrees-of-freedom (DoF) in the motion control, the quadruped robots show good gait stability and controllability compared to any other robotic systems. For examples, Boston Dynamics introduced Big Dog and LS3, which are able to walk on uneven terrain at the speed of about 4.4 miles per hour [3,4,5], and SpotMini, which can autonomously navigate and move in general areas, such as flat ground, rough terrain, office/construction sites, passing through steps, and avoiding human/obstacles [6,7,8]. MIT introduced the MIT Cheetah series, which are quadruped robots inspired by a cheetah, which is equipped with directly driven actuators [9]. MIT Cheetah shows a broad range of leg movement and more focus on versatility [10,11], and MIT Mini Cheetah is capable of acrobatic motions, such as a back-flip, in addition to all the capabilities of its predecessor [12,13]. Additionally, the Korea Institute of Industrial Technology developed a quadruped robot actuated by hydraulic actuators, which is able to walk at about 3.4 miles per hour with a 60 kg load [14].

For the locomotion of the quadruped robot, various trajectory generation methods have been studied. For example, a central pattern generator (CPG) is often utilized to solve this problem. The CPG consists of coupled nonlinear dynamic equations, which are usually formulated in a neural network framework. The CPG generates rhythmic control policies for the locomotion of the quadruped robot [15]. Crespi et al. utilized the CPG to generate the locomotion of a robot inspired by a snake [16]. Another approach trajectory generation method for the robotic system is a finite states machine (FSM). FSM is a computational model of the machine with an initial internal memory that defines some finite states for the machine. Liu et al. applied the FSM for the separation of the swing phase and the stance phase [17]. Hussain et al. elaborated and tested the functional structure of a control system using the logic-labeled FSM [18], and Ding et al. generated the trotting, bounding, and aperiodic motions by event-based FSM that was extended from time-based FSM through the contact detection algorithm [19].

However, these conventional trajectory generation methods (i.e., CPG, FSM, etc.) have limited degrees of freedom, because gait phases (i.e., walk, amble, trot, canter, and gallop) of the quadruped system is changed very frequently according to the gait speed. Quadruped animals effectively accelerate and decelerate the gait speed by changing their gait phases. Since quadruped animals have been evolved over millions of years, the gait phases and the associated leg motions of quadruped animals can be regarded as optimal motion for the locomotion of the quadruped animals. For example, the walk is an optimized leg motion for low-speed locomotion, and the gallop is optimized for high-speed locomotion. The efficiency of the gait phases of quadruped animals was investigated by many biologists [20,21]. Therefore, it is reasonable that the trajectory generation for the quadruped robot should realize multiple gait phases to handle the locomotion speed of a wide range. Furthermore, to efficiently change the speed of the quadruped robot, the gait phase transition should be realized smoothly.

Since the conventional trajectory generation methods have been either entirely or partly based on pre-programmed network topologies, the gait transition from low to high-speed locomotion is not possible in the conventional trajectory generation methods. Furthermore, the conventional trajectory generation methods do not consider the realization to a sufficient degree, leading to pattern generation that neither self-organizes nor is efficient in response to real-world scenarios. Although adaptive trajectory generation methods have been developed to change gait phases adaptively and to qualitatively describe the behavior of animals, the number of parameters that can be adjusted may still be limited [22]. Moreover, the result of the trajectory generation, which is the limit cycle of the coupled nonlinear dynamic functions, is not fully adaptable. Another challenge in the use of the conventional trajectory generation methods is stability; the stability of synchronization between the result of the trajectory generation is challenging to prove in general. Therefore, the conventional trajectory generation methods may not be appropriate for the quadruped robot. That is why the general quadruped robots that have been developed and announced do not consider the transition of the gait phase or use a pre-determined fixed gait phase. The SpotMini of the Boston Dynamics, which is known to be the best in this field, also had pre-determined gait phases (walk and trot) applied, and the transition of the gait phase has to be completed before the start of the locomotion [23]. Although some research teams have developed trajectory generation algorithms that take into account the transition of the gait phase, the transition of the gait phase is limited (mostly from walking to trot), and the transition according to gait speed is not considered [24,25].

In this paper, a multi-phase joint-angle trajectory generation algorithm is proposed for the quadruped robot. The main proposed ideas of the multi-phase joint-angle trajectory generation algorithm are (1) there exist the cyclic basis functions of the joint-angle trajectories of the quadruped animal; (2) there exists a unique ground contact sequence for each gait phase (i.e., walk, amble, trot, canter, transverse gallop, rotatory gallop) according to the gait speed; (3) quadruped animals effectively accelerate and decelerate the locomotion speed by smooth gait transition in their gait phases; and (4) these ideas have to be considered to generate the joint-angle trajectories for the quadruped robots. To realize these proposed ideas, the joint-angle trajectories for the control of the quadruped robot in multiple gait phases are designed by a single cyclic basis function, which is designed based on the experimental results with a dog. The input to the basis function is controlled to realize desired trajectories using a static synchronization function, and the synchronization function is formulated by which the motions of legs are designed to have proper ground contact sequences (there exists a unique ground contact sequence for each gait phase, which has been typically used to distinguish the gait phases of quadruped animals [26]) with each other. Furthermore, to realize the smooth gait transition according to the gait speed, a fuzzy logic method is applied to the proposed method. The desired ground contact sequence for each gait phase is determined according to the literature in [27,28], and they are formalized as a time-varying vector with respect to the gait speed using fuzzy logic.

This paper is organized as follows. In Section 2, studies on the gait of the quadruped animal, particularly the ground contact sequence on each gait phase and characteristics on the stance and swing periods, are explored. In Section 3, the proposed gait phase generation algorithm is introduced. In addition, the reference joint-angle trajectories inspired by the quadruped animal are introduced, and the trajectories are represented as a cyclic basis function. In Section 4, the ground contact sequences are interpolated smoothly by fuzzy logic with respect to the gait speed. Then, the ground contact sequence is transformed into a desired input of a cyclic basis function using a static synchronization function. Lastly, the desired joint-angle trajectories are decided using the cyclic basis function, and the developed algorithm is discretized for implementation to the simulation. In Section 5.2, the simulation result of the proposed algorithm is shown. Finally, Section 6 renders a summary and future works.

## 2. Gait of Quadruped Animal

### 2.1. Experiment Settings

**Figure 1:**
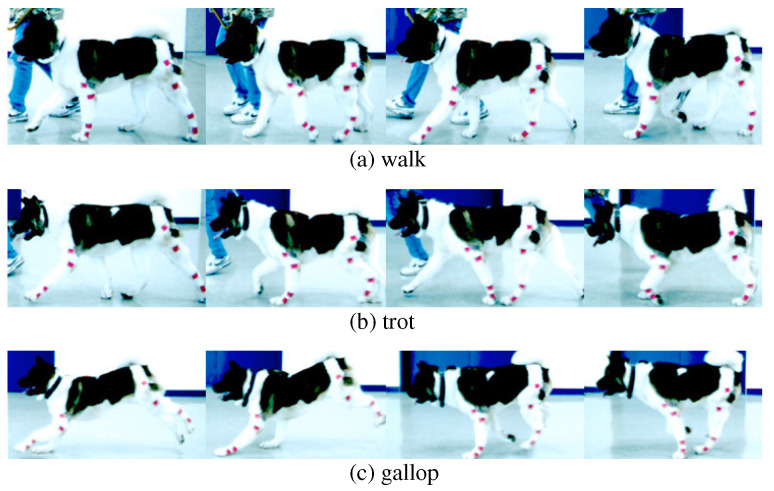
Observation of the gait of a dog in (**a**) the walk phase, (**b**) the trot phase, and (**c**) the gallop phase.

Success in the realization of gait motions of the quadruped robot is highly dependent on the adequacy of generated joint-angle trajectories, as well as the mechanical performance of actuators, the power capacity, and the performance of control algorithms. The joint-angle trajectories of the quadruped robot are complicated and different according to gait phases, which creates a challenge in their mathematical derivation. Therefore, in this paper, joint-angle trajectories for the control of the quadruped robot are inspired by and designed from the joint-angles of an animal that runs fast exhibiting various gait phases, as shown in Figure 1. The gait motions of an animal can be regarded as optimal motions for effective locomotion of the animal. Therefore, observation of the animal’s gait motions may provide fundamentals for the realization of the successful locomotion of a quadruped robot. While quadruped animals walk or run, the joint-angle trajectories exhibit certain patterns on each gait cycle. The joint-angle patterns may provide fundamentals in the control of the joints of the quadruped robot, i.e., the joint-angles of the quadruped animal may be utilized as the reference input for feedback control.

The joint-angle trajectories are obtained from experiments with a fast-running dog in this paper. The species of the dog used in the experiments is American Akita, which is regarded as one of the fastest dogs; American Akitas exhibit the multiple gait phases, as shown in Figure 1, and are able to run at the gait speed of forty kilometers per hour.

During the gait of the quadruped animals, the whole body segments (e.g., the tail, the head, and so on) are actively involved. For example, it is well-known that the tail has a function to maintain the balance of the body in high-speed locomotion. Nevertheless, the four legs are still major components among the body segments that realize the gait, and thus, the leg motions are mainly analyzed in the experiments for the design of a trajectory generation algorithm of a quadruped robot. In addition, since the leg motions during high-speed locomotion are mainly on the sagittal plane, the joint-angle trajectories on the sagittal plane are considered in this paper.

**Figure 2:**
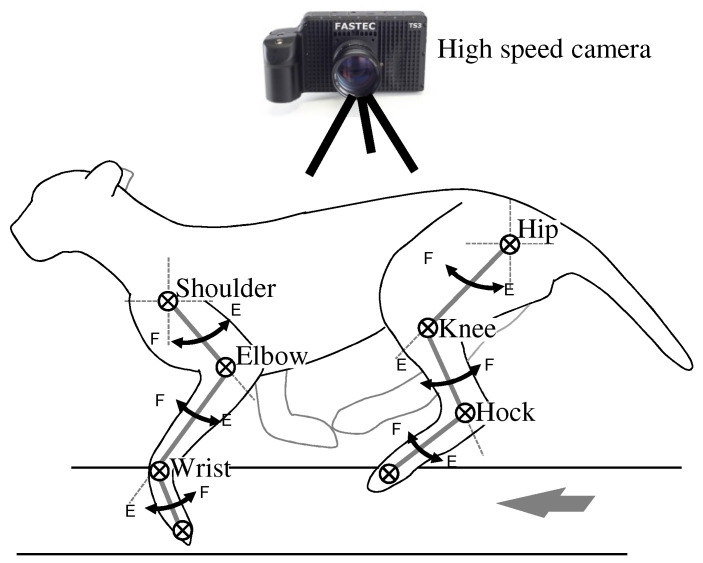
An experimental setting and the direction of joint-angle measurements.

The experimental setting is shown in Figure 2. To observe the gait of the dog from experimental results, the joint-angles should be measured. For this purpose, a high-speed camera, TS3 of Fastec Co. [29], which takes a video at 98,000 frames per second, was utilized. In order to obtain the absolute positions from the projected images obtained by the high-speed camera, red markers were attached to the locations of the dog’s joints, as shown in Figure 1; the locations of the red markers are also marked as ⨂ in Figure 2. Since the camera takes only a set of two-dimensional images, the camera was installed far enough, and the dog was forced to run on a straight line by a trainer. From each frame of videos, the absolute positions of the red markers were extracted, and the joint-angles were calculated by arc tangent functions.

Considering that the four legs are the main components that generate locomotion, the majority of gait motions can be described by the twelve joint-angles (i.e., three joint-angles per leg), which are references for controlling a quadruped robot. Since the motions of the left and right legs share the same characteristics, and those of the front legs and the hind legs show significantly different characteristics due to the mechanical configuration, the proposed algorithm generates six unique joint-angle patterns, i.e., three joint-angle trajectories for the front leg, and three joint-angles for the hind leg. The joint-angle trajectories of the left and right legs can be generated by shifting the trajectories with an appropriate time-interval. Therefore, in this paper, the characteristic patterns of the six joints of the dog are observed and analyzed for formulating reference angles. The measurements of the joint-angles of the dog follow the general rule in the anthropometry except on the hip and shoulder joints. They are measured based on the vertical line for the sake of simplicity. In Figure 2, F and E represent the directions of flexion and extension, respectively.

### 2.2. Joint-Angle Measurements

**Figure 3:**
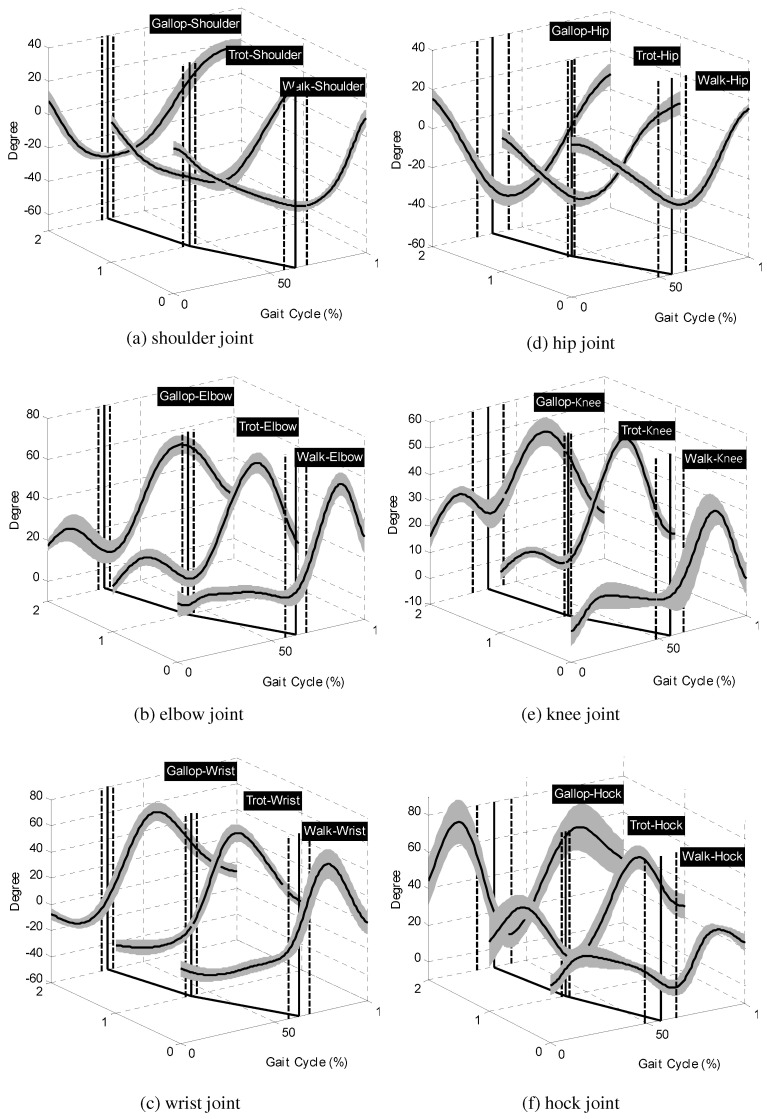
Joint-angle trajectories of a dog in multiple gait phases: (**a**) the shoulder joint-angle, (**b**) the elbow joint-angle, (**c**) the wrist joint-angle, (**d**) the hip joint-angle, (**e**) the knee joint-angle, and (**f**) the hock joint-angle. The black continuous lines and the gray bands represent the mean values and the standard deviation of measurements, respectively. The numbers in the *y*-axis mean 0: walk phase, 1: trot phase, and 2: gallop phase.

Figure 3 shows the obtained joint-angles of a dog in multiple gait phases. Since the period of one gait cycle, i.e., the time-interval between heel-strikes for each leg, varies according to the gait speed, the joint-angles shown in the figure were normalized to a gait cycle. Moreover, the period of a gait cycle should be multiplied to the percentage of the gait cycle in order to obtain the joint-angle information in the actual time domain. It should be noted that a unique pattern is observed in each joint-angle trajectory shown in Figure 3. For example, the shoulder and hip joints exhibit pendulum motions regardless of the gait phase, and the elbow joints show major flexion during swinging and minor flexion during stance. The similar patterns are also shown in the wrist joint angles, the knee joint, and the hock joint angles. Therefore, the observed joint-angles can be formalized into a representative function. However, the amplitudes and patterns of the joint-angle trajectories still show large variations as the gait speed changes, which creates a challenge in the derivation of a representative function.

### 2.3. Stance and Swing Periods

The period that a foot touches the ground is called the stance, and the period that the animal moves the foot in the air is called the swing. In Figure 3, the gait cycle starts with the stance period, and the swing period starts at the location of the continuous vertical line shown in the figures. The dashed vertical lines around the continuous vertical lines represent the standard deviation for each joint angle in each gait phase. The detailed analysis results on the observed leg motions of the dog are shown in Table 1.

For the generalization of the obtained data, the gait speed was normalized with the body length of the dog. Since the body length of the dog from the shoulder to the hip is 0.6 (m), the actual gait speed can be obtained by multiplying this number. As the gait speed increases, the stance period is reduced, which implies that the time that the corresponding foot is in contact with the ground is shortened. On the other hand, it should be noted that the change of the swing period is minor (see the values of the Swing period row in Table 1). This is because (1) gait stability is achieved mainly by stance legs, and (2) the swing period is minimal for the rapid recovery of the leg position. Since the standard deviation of the swing periods were 0.02∼0.09 s, it is reasonable to assume that the swing period is constant regardless of the gait speed and phase. Consequently, the average ratio of the stance period is decreased, as the gait speed increases.

### 2.4. Joint-Angles in a Scaled Gait Cycle Domain

For the formalization of the obtained joint-angles in multiple gait phases, the time axes of the joint-angle trajectories are scaled. It should be noted that the characteristic patterns are shown in the joint-angle trajectories of the stance and swing periods, respectively. For example, in the elbow joint-angles, although their shapes on the actual time domain are different according to the gait speeds, the major flexion is observed in every swing phase, and the minor flexion is shown in every stance phase. Therefore, the time axes of the obtained joint-angles are scaled such that [0,50)% of the gait cycle is the stance period, and the remaining [50,100)% of the gait cycle is the swing phase.

**Figure 4:**
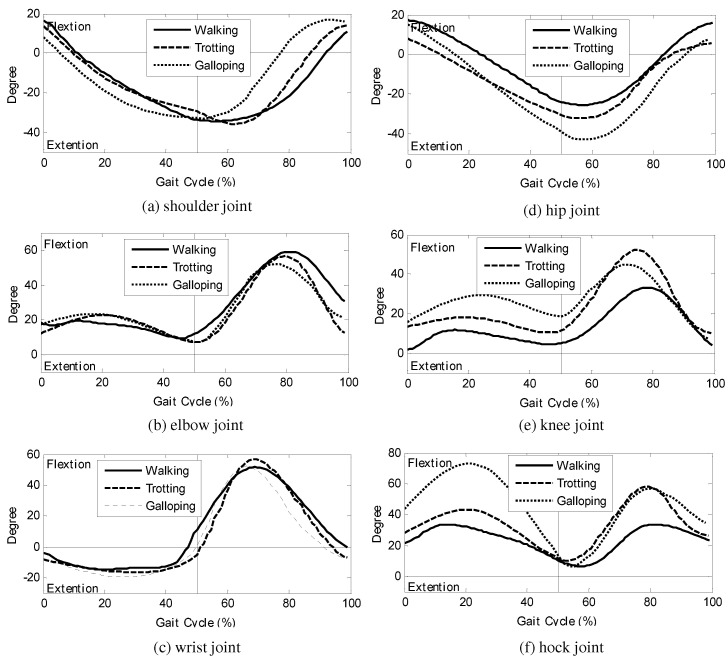
Joint-angle trajectories over scaled gait cycles: (**a**) the shoulder joint-angle, (**b**) the elbow joint-angle, (**c**) the wrist joint-angle, (**d**) the hip joint-angle, (**e**) the knee joint-angle, and (**f**) the hock joint-angle.

The joint-angle trajectories of the dog on the scaled gait cycle domain are shown in Figure 4. It should be noted that the shapes of the joint-angle trajectories, as well as their patterns, match for all the gait phases. Therefore, the joint-angle trajectories on the scaled gait cycle domain can be formulated by a single represented function, named a *cyclic basis function* in this paper.

## 3. Formulation of Joint-Angle Trajectory

### 3.1. A Cyclic Basis Function

It should be noted that the gait cycle is scaled in Figure 4 in order to formulate the joint-angle trajectories at different gait speeds into a single representative function (i.e., one cyclic basis function for each joint). When the gait cycle is scaled such that the stance and the swing sections are divided equally, the shapes of the joint-angle trajectories match, which can be represented by a single cyclic basis function. Any function is applicable for the cyclic basis function, as long as it is continuous and differentiable in the gait cycle domain:

(1) $$\theta_{i}=f_{i}\left(\phi \right)$$

where i=1,2,…,6, where each number represents the shoulder, the elbow, the wrist, the hip, the knee, and the hock joints. ϕ is the index on the scaled gait cycle domain. In order to emphasize the cyclic nature of the joint-angle trajectories, trigonometric functions are utilized for the formalization of the joint-angles, i.e.,

(2) $$f_{1}\left(\phi \right)=-0.3+s_{F}\left[0.4\cos(\phi )+0.1\cos(2\phi +0.6)\right]$$

(3) $$f_{2}\left(\phi \right)=0.45+s_{F}\left[0.3\cos(\phi +1.1)+0.2\cos(2\phi +2.9)\right]$$

(4) $$f_{3}\left(\phi \right)=0.1+s_{F}\left[0.6\cos(\phi +1.8)+0.2\cos(2\phi -2.3)\right]$$

where $s_{F}$ is a scaling factor that determines the amplitudes of joint-angle trajectories. The cyclic basis functions for the hind leg are

(5) $$f_{4}\left(\phi \right)=-0.2+s_{H}\left[0.24\cos(\phi -0.2)+0.03\cos(2\phi +0.9)\right]$$

(6) $$f_{5}\left(\phi \right)=0.4+s_{H}\left[0.1\cos(\phi +1.7)+0.1\cos(2\phi -2.9)\right]$$

(7) $$f_{6}\left(\phi \right)=s_{H}\left[0.35+0.15\cos(\phi -0.2)+0.16\cos(2\phi +2.9)\right]$$

where $s_{H}$ is a scaling factor. Figure 4 shows the cyclic basis functions in Equations (2)–(7). Notice that the designed functions represent the patterns and shapes of the joint-angles in multiple gait phases.

**Figure 5:**
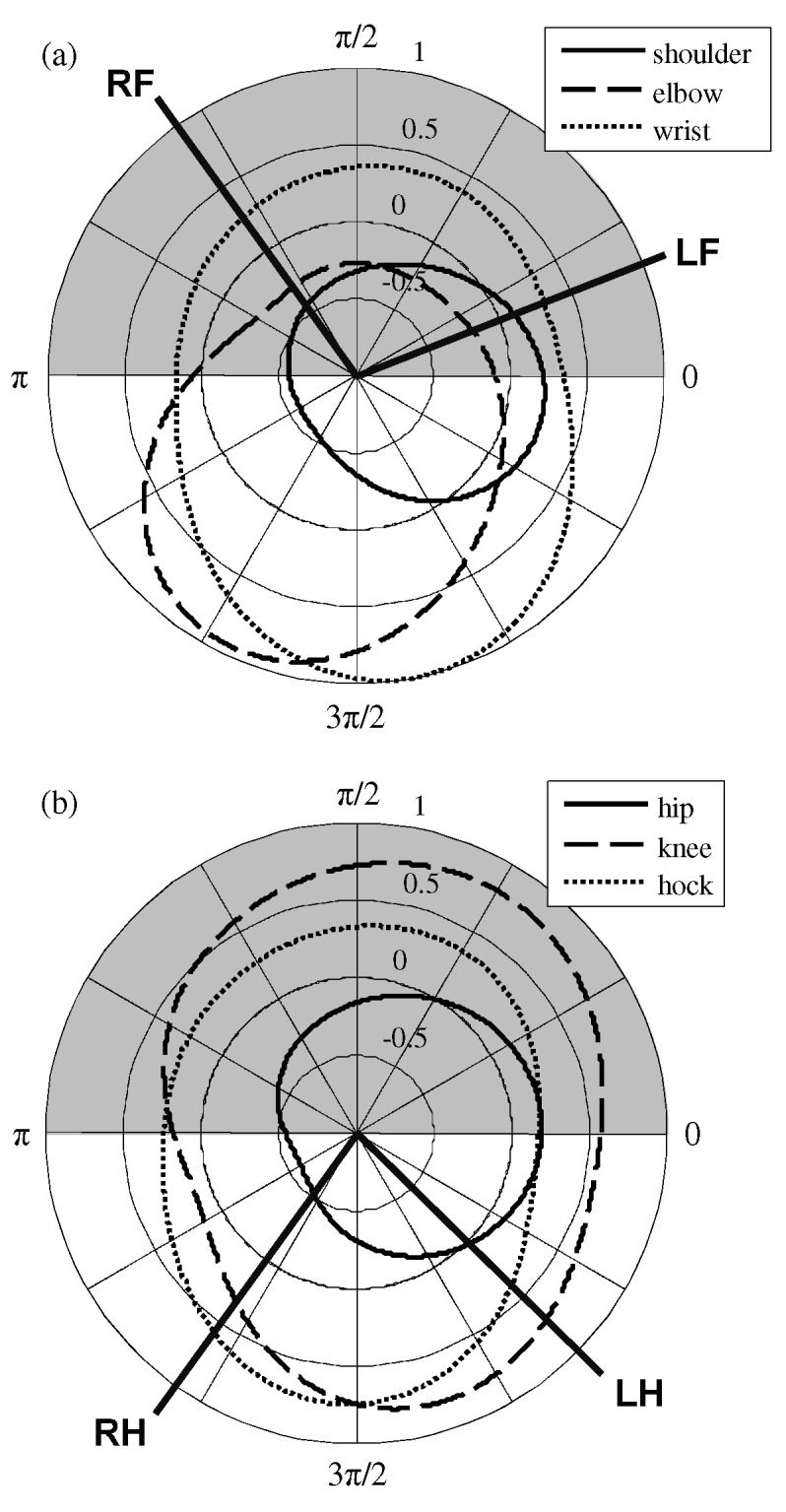
Cyclic basis functions in the *r*-ϕ coordinate system: (**a**) front leg and (**b**) hind leg.

Notice that the cyclic basis functions in Equations (2)–(7) are cyclic (i.e., $f_{i}\left(\phi \right)=f_{i}\left(\phi +2\pi n\right)$ for any integer number *n*) and differentiable for ϕ. Such characteristics are only valid if the scaled gait cycle is further scaled into the range of [0,2π). Since the scaled gait cycle in Figure 4 divides the stance period and the swing period equally, [0,π) corresponds to the stance period, and [π,2π) represents the swing period in the scaled gait cycle domain. The cyclic basis functions are to generate cyclic and continuous joint-angle trajectories as ϕ increases continuously. For a better representation of the cyclic nature, the cyclic basis functions can also be represented in the *r*-ϕ coordinate system, as shown in Figure 5, where the radii at an angle (ϕ) are the values of the joint-angles in the scaled gait phase. The gray area in Figure 5 represents the stance period.

### 3.2. Scaling Factor

As the gait speed increases, the shape of the joint-angle trajectories remains the same in the scaled gait cycle domain, but their magnitudes change. Notice that scaling factors are considered in the cyclic basis functions in Equations (2)–(7), where $s_{F}$ is a scaling factor for the three joint-angles of the front leg, and $s_{H}$ is one for the three joint-angles of the hind leg. As the gait speed increases, the range of joint-angles is increased, which may require increasing the scaling factors according to the gait speed. Through the experimental results, it was observed that the amplitudes of the hind leg motions are increased proportionally, as the gait speed increases. Therefore, the scaling factors, $s_{F}$ and $s_{H}$, are formulated as

(8) $$s_{F}=q_{F}v\left(t\right)+1$$

(9) $$s_{H}=q_{H}v\left(t\right)+1$$

where v(t) is the gait speed in the unit of meter per second, and $q_{F}$ and $q_{H}$ are constants. In the case of the dog used in the experiments, $q_{H}=0.3$ and $q_{F}=0.0$, which means that the amplitudes of the hind leg motions change according to the gait speed, but those of the front leg motions are constant regardless of the gait speed.

### 3.3. Ground Contact Sequences

**Figure 6:**
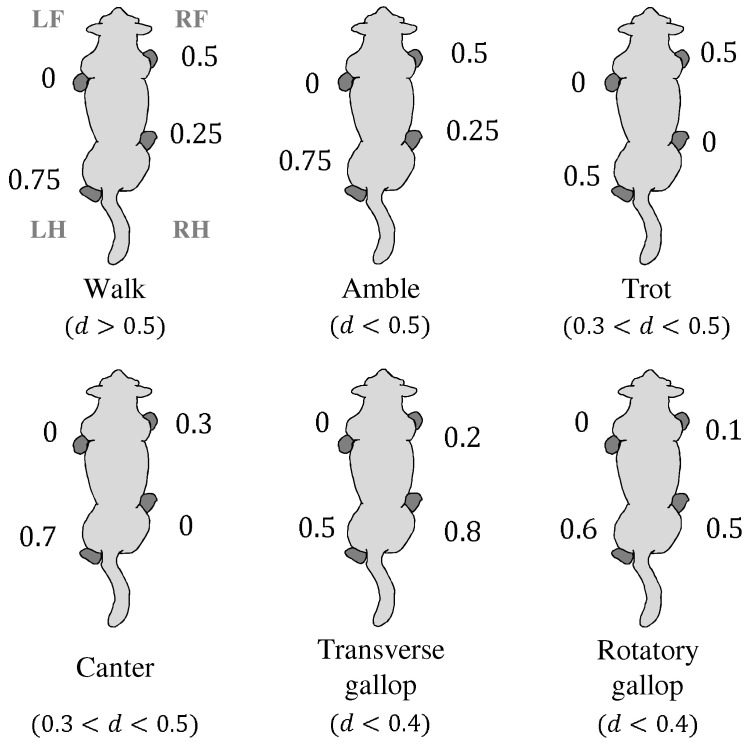
Ground contact sequences with respect to gait phases; LF, LH, RF, and RH refer to the left-front, the left-hind, the right-front, and the right-hind, and *d* means the continuous ground contact time ratio of one gait cycle period.

The quadruped locomotion is a combination of motions of the four legs. While the joint-angles of each leg can be analyzed and simulated by the corresponding cyclic basis functions, the ground contact sequences and their time-intervals should also be considered for the generation of overall locomotive motions. The ground contact sequences and time-intervals vary according to the gait phase. Namely, there exists a characteristic ground contact sequence defined for each gait phase (e.g., walk, trot, canter, gallop, etc.), as shown in Figure 6 [26], and they should be effectively considered in the joint-angle trajectory generation.

In the ground contact sequences shown in Figure 6, the intervals between ground contact instances are determined with respect to the LF leg, and thus, the intervals for the LF leg are all zero. The intervals shown in the figure are normalized with a gait cycle period; the period of one cycle may be multiplied to the numbers shown in the figure to obtain the actual time-intervals. The normalized intervals are used as the reference intervals of the proposed gait motion generation algorithm and are denoted as $R_{d}$, i.e.,

(10) $$R_{d}=\left[\begin{array}{cccc} R_{d}^{lf} & R_{d}^{lh} & R_{d}^{rf} & R_{d}^{rh} \end{array}\right]\in R^{4\times 1}$$

The second component, $R_{d}^{lh}$, means the normalized time-interval between the LF leg and the LH leg. Similarly, $R_{d}^{rf}$ and $R_{d}^{rh}$ mean the normalized time-intervals between the LF and the RF leg and the RH leg, respectively. The values of the components of $R_{di}$, where i=1,2,…,5 represents each gait phase, is listed in Table 2. Remember that the intervals in Table 2 are normalized values, as in Figure 6; thus, the period of one gait cycle should be multiplied to the values. For example, assuming that the period of one gait cycle is $T_{cycle}$ seconds, and the gait phase of a robot is in a walking phase, the locomotion of a robot is to be controlled such that the LF foot touches the ground first, and the remaining three feet touch the ground with the time-interval of $0.25T_{cycle}$ seconds in the sequence of RH, RF, and LH feet.

Notice that the reference intervals, $R_{di}$s, shown in Table 2, are defined for the five phases; the walk, trot, canter, transverse gallop, and rotatory gallop. The amble phase in Figure 6 is not taken into account in $R_{di}$s because the normalized intervals of the amble phase are the same as the walk phase, and the period of the amble phase is too short that it is negligible in the locomotion of typical quadruped animals.

## 4. Joint-Angle Trajectory Generation for Quadruped Robots

### 4.1. Gait Transition by Fuzzy Logic

Although the definitions of gait phases are discrete, as in Table 2, the gait speed of actual animals continuously varies over time, and their leg motions are smooth and continuous. In a robotic system, the smooth and continuous leg motions are also desired for natural and effective locomotion. Otherwise, the abrupt change of joint-angle trajectories may cause actuator saturations and instability of the gait.

In the proposed algorithm, the reference (i.e., desired) ground contact sequences for each gait phase is $R_{di}$. The discrete values should be effectively interpolated with each other, such that the value of every component of a resultant reference ground contact sequence, $R_{df}\left(v\right)$, is smooth and continuous (i.e., differentiable for the time). For this purpose, a Mamdani type fuzzy logic system is applied to the proposed algorithm [30,31,32].

**Figure 7:**
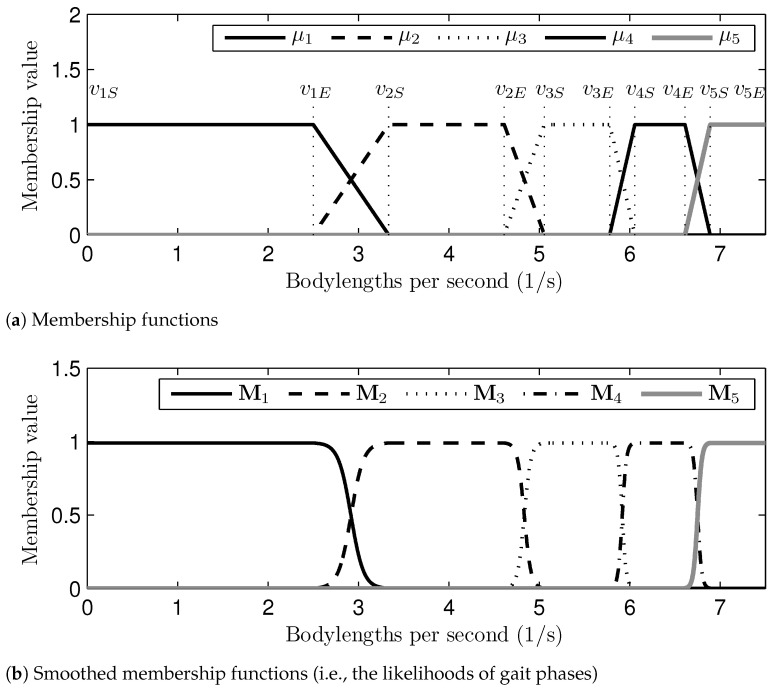
Transition of gait phases by fuzzy logic: (**a**) membership functions and (**b**) smoothed membership functions (i.e., the likelihoods of gait phases).

The characteristic speeds that distinguish the desired gait phases may vary according to the species of quadruped animals. For example, the locomotion of the dog used in the experiments was definitely in the walk phase at the speed of 1.19 (m/s) (i.e., 1.98 bodylength per second), the trot phase at the speed of 2.69 (m/s), and the gallop phase at the speed of 4.34 (m/s), as shown in Table 1. Namely, if the gait speed is the same as one of the characteristic speeds, then the desired gait phase can be determined deterministically. However, the transition of the gait phases may occur in between the characteristic gait speeds. In this paper, the transition of gait phases is realized by the linear interpolation of two gait phases adjacent to the current gait speed. Following this logic, the likelihoods of the gait phases are modeled as membership functions (i.e., $\mu_{i}$, where i=1,2,…,5 means each gait phase: walk, trot, canter, transverse gallop, and rotary gallop), as shown in Figure 7a. $v_{iS}$ is the starting point of the likelihood changing at each gait phase, and $v_{iE}$ is the ending point of the likelihood changing at each gait phase.

(11) $$v_{iS}=v_{i}-r\left(v_{i}-v_{i-1}\right)$$

(12) $$v_{iE}=v_{i}+r\left(v_{i+1}-v_{i}\right)$$

where *r* representatives the changing ratio of the likelihood. In this condition, the membership functions of each gait phase are defined as

(13) $$\mu_{i}\left(v\right)=\left\{\begin{array}{cc} \frac{1}{v_{i-1,E}-v_{iS}}\left(v-v_{i-1,E}\right)+1, & \mathrm{for}v_{i-1,E}\leq vs.\leq v_{iS}\\ 1, & \mathrm{for}v_{iS}\leq vs.\leq v_{iE}\\ \frac{1}{v_{i,E}-v_{i+1,S}}\left(v-v_{iE}\right)+1, & \mathrm{for}v_{iE}\leq vs.\leq v_{i+1,S} \end{array}\right.$$

The magnitudes of the membership functions represent the likelihood of each gait phase. For example, at the gait speed of 0.5 bodylength per second, $\mu_{1}=1$ and $\mu_{2,3,4,5}=0$, which implies that the desired gait phase is definitely the walk phase. On the other hand, if the gait speed is nearly by 3 bodylength per second, $\mu_{1,2}=0.5$ and $\mu_{3,4,5}=0$, it means that the likelihoods of both the walk and trot phases are the same. Notice in the figure that the characteristic speeds are represented as ranges, i.e., $\mu_{i}=1$ and $\mu_{j\neq i}=0$ if $v\left(t\right)\in \left[v_{iS},v_{iE}\right]$ for i=1,2,…,5.

The membership functions for modeling the likelihoods of gait phases, however, are not smooth. Since the proposed algorithm changes the gait phases (i.e., gait transition) by changing the membership functions based on the fuzzy logic, the non-smooth characteristic of the membership functions can cause a drastic change in the ground contact sequence. This is the main problem of gait stability, such as dynamics change, control performance, and so on. Therefore, the membership functions have to generate as smoothly as possible. For further smoothing of the membership functions, new membership functions are defined as functions of $\mu_{i}\left(v\right)$’s, i.e.,

(14) $$M_{i}\left(v\right)=\frac{0.5\left[\tanh\left(s(\mu_{i}\left(v\right)-0.5)\right)+1\right]}{\sum_{i=1}^{5}M_{i}\left(v\right)}$$

where i=1,2,…,5 is the gait phase index, which is the same as in $\mu_{i}$’s, and $s\in R_{+}$ is a sensitivity factor that changes the slope of the membership functions in transition. The shapes of $M_{i}\left(v\right)$s are represented in Figure 7b. If *s* is too small, the deterministic section (i.e., the section where $M_{i}\left(v\right)=1$) does not appear. On the other hand, if *s* is too big, the phase transition becomes too drastic. Thus, an appropriate *s* should be used; an appropriate value for *s* was empirically found to be 6.0. It should be noted in Equation (14) that $M_{i}\left(v\right)$ is normalized by the sum of all $M_{i}\left(v\right)$s, which is to make the sum of all $M_{i}\left(v\right)$s one in any situation. In the proposed algorithm, $M_{i}\left(v\right)$ is regarded as the likelihood of *i*th gait phase.

Since the total likelihood of gait phases (i.e., $\sum_{i=1}^{5}M_{i}\left(v\right)$) is always one, $M_{i}\left(v\right)$s can be used as weighting factors for the calculation of the desired ground contact sequence, i.e.

(15) $$\begin{array}{c} R_{df}\left(v\right)=\sum_{i=1}^{5}R_{di}M_{i}\left(v\right)\in R^{4\times 1} \end{array}$$

Since $M_{i}\left(v\right)$ returns a value close to one in the characteristic speed range, $R_{df}\left(v\right)={R_{d}}_{i}$ for $v\left(t\right)\in \left[v_{iS},v_{iE}\right]$. When $v\left(t\right)\notin \left[v_{iS},v_{iE}\right]$, i.e., the gait phase is not deterministic, $M_{i}\left(v\right)$s smoothly interpolate the values of ${R_{d}}_{i}$s adjacent to the given gait speed.

### 4.2. Synchronization of Leg Motions

The inputs of the cyclic basis functions in Equations (2)–(7) are controlled to generate appropriate joint-angle trajectories with desired ground contact sequences obtained in the previous section. Three different cyclic basis functions are applied for the three joints of a leg (i.e., Equations (2)–(4) for the front leg, and Equations (5)–(7) for the hind leg), while the inputs to the three cyclic basis functions are identical for each leg. The input of a cyclic basis function is an index on the scaled gait cycle domain, and thus, the locomotion of four legs is realized by determining four indexes on the scaled gait cycle domain, i.e.,

(16) $$\Phi \left(t\right)={\left[\begin{array}{cccc} \phi^{lf}\left(t\right) & \phi^{lh}\left(t\right) & \phi^{rf}\left(t\right) & \phi^{rh}\left(t\right) \end{array}\right]}^{T}\in R^{4\times 1}$$

where each component is the index on the scaled gait cycle domain for each leg, i.e., the input of the cyclic basis functions. The components of Φ(t) should be effectively updated considering the desired ground contact sequences and the gait speeds. Namely, the adequacy of the joint-angle trajectories generated by the proposed algorithm is dependent on how the components of Φ(t) are effectively updated.

In order to reflect the desired ground contact sequences in the calculation of Φ(t) in real-time, the desired time-intervals of ground contact instances (i.e., the moments of heel-strike) should be obtained first. Remember that the desired ground contact sequence, i.e., $R_{df}\left(v\right)$ in Equation (15), includes the desired intervals normalized to one gait cycle. Therefore, the actual time-intervals are obtained by multiplying the total period of one gait cycle to $R_{df}\left(v\right)$, i.e.,

(17) $$\left[\begin{array}{c} \tau^{lf}\left(v\right)\\ \tau^{lh}\left(v\right)\\ \tau^{rf}\left(v\right)\\ \tau^{rh}\left(v\right) \end{array}\right]=T_{cycle}\left(v\right)R_{df}\left(v\right)\in R^{4\times 1}$$

where $T_{cycle}\left(v\right)$ is the total period of one gait cycle. Since the first component of $R_{df}\left(v\right)$ is always zero, the time-interval for the LF leg, $\tau^{lf}\left(v\right)$, is zero. Remember that the total period of one gait cycle varies according to the gait speed, as shown in Table 1. The total period of one gait cycle consists of periods of the stance ($T_{st}\left(v\right)$) and the swing ($T_{sw}$), i.e.,

(18) $$T_{cycle}\left(v\right)=T_{st}\left(v\right)+T_{sw}$$

**Figure 8:**
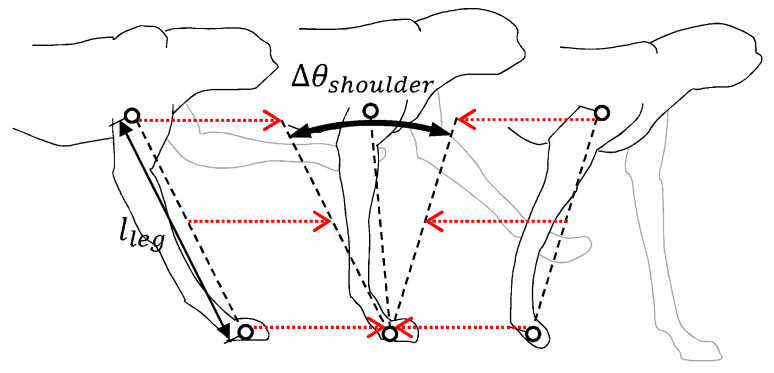
The locomotion of the front-leg during the stance period.

As discussed in Section 2.3, the period of the swing is constant regardless of gait phases and speeds. In the case of the dog used in the experiments of this paper, $T_{sw}=0.34$ s. On the other hand, the period of the stance is reduced as the gait speed increases. Noting that the body moves forward during the stance period, as shown in Figure 8, $T_{st}\left(v\right)$ can be calculated as

(19) $$T_{st}\left(v\right)=\frac{l_{leg}\Delta \theta_{e}}{v}$$

where $l_{leg}$ is the length of a leg (i.e., the distances between the wrist and shoulder joints and the hock and hip joints), and $\Delta \theta_{e}$ is an effective angle change during the stance period. Since the angle variation of the shoulder and hip joints are dominant in the stance period, $\Delta \theta_{e}$ can be regarded as $\Delta \theta_{shoulder}$ and $\Delta \theta_{hip}$ for the sake of simplicity.

**Figure 9:**
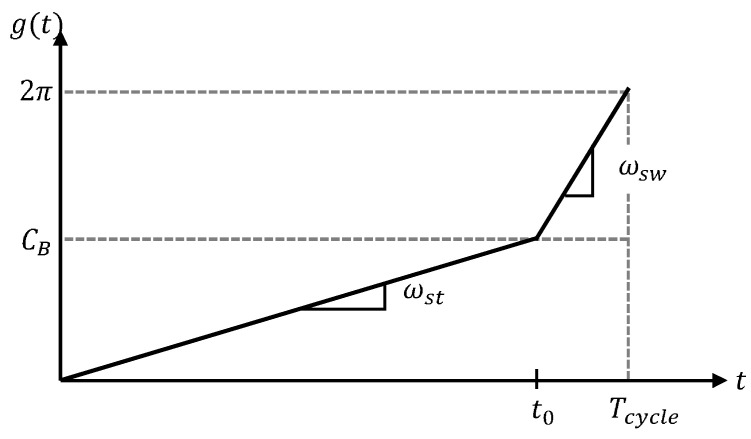
Rates of the input of cyclic basis functions; $t_{0}=\omega_{st}^{-1}\pi$, $\omega_{sw}$ and $\omega_{st}$ are the rates in the swing and stance periods, and $T_{cycle}$ means the total period of one gait cycle.

Once the total period of one gait cycle is obtained as in Equation (18), the input of the cyclic basis functions can be calculated. Remember that the cyclic basis functions are on the domain of the scaled gait cycle. Therefore, the rate of the scaled gait cycle index in the stance period (i.e., [0,π) on the scaled gait cycle domain) may be different from that in the swing period (i.e., [π,2π) on the scaled gait cycle domain). The two rates are defined as

(20) $$\begin{array}{ccc} \omega_{st}\left(v\right) & = & \frac{\pi }{T_{st}\left(v\right)} \end{array}$$

(21) $$\begin{array}{ccc} \omega_{sw} & = & \frac{\pi }{T_{sw}} \end{array}$$

where $\omega_{st}\left(v\right)$ means the rate of ϕ(t) in the stance period, and $\omega_{sw}$ is the rate in the swing period. Figure 9 depicts how the index of the scaled gait cycle domain is updated in the actual time domain. It can also be formalized as

(22) $$g\left(t\right)=\left\{\begin{array}{cc} \omega_{st}\left(v\right)t, & \mathrm{for}0\leq t<t_{0}\\ \omega_{sw}\left[t-t_{0}\right]+\pi , & \mathrm{for}t_{0}\leq t<T_{cycle}\left(v\right) \end{array}\right.$$

where $t_{0}=\omega_{st}^{-1}\pi$. Since the gait motions are cyclic, g(t) should also be cyclic with the period of $T_{cycle}\left(v\right)$, i.e.,

(23) $$g(t+nT_{cycle}\left(v\right))=g\left(t\right)$$

where *n* is any integer number.

The four indexes in the scaled gait cycle domain are updated by Equation (22) with the desired time-intervals in Equation (17). Therefore, the inputs of the cyclic basis functions are calculated as

(24) $$\left[\begin{array}{c} \phi^{lf}\left(t\right)\\ \phi^{lh}\left(t\right)\\ \phi^{rf}\left(t\right)\\ \phi^{rh}\left(t\right) \end{array}\right]=\left[\begin{array}{c} g\left(t+\tau^{lf}\left(v\right)\right)\\ g\left(t+\tau^{lh}\left(v\right)\right)\\ g\left(t+\tau^{rf}\left(v\right)\right)\\ g\left(t+\tau^{rh}\left(v\right)\right) \end{array}\right]$$

### 4.3. Discretization for Implementation

**Figure 10:**
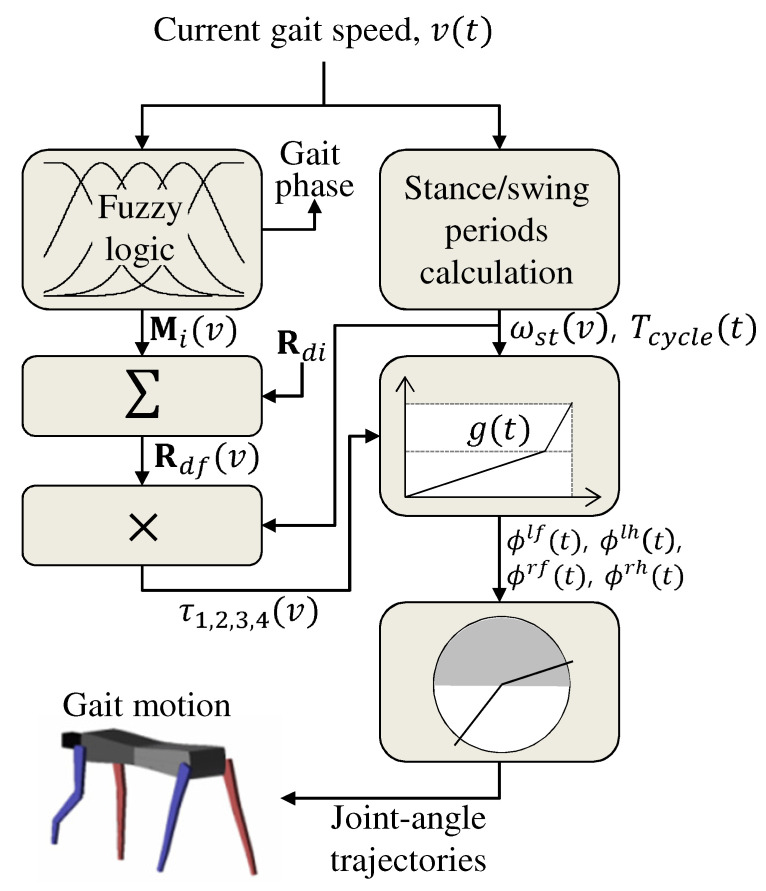
A block diagram of the proposed algorithm.

The overall procedure of the proposed joint-angle trajectory generation algorithm is shown in Figure 10. Firstly, for a given (i.e., current) gait speed, $R_{df}\left(v\right)$ is obtained by interpolating the desired ground contact sequences defined for each gait phase, $R_{di}$. A fuzzy logic method is applied for a smooth and continuous transition of the gait phases. Then, the rates of the scaled gait cycle indexes are calculated for each leg, as in Equation (22). With the calculated rates, the inputs of the cyclic basis functions are updated with the desired time-intervals. Finally, the joint-angle trajectories are obtained by the cyclic basis functions for each joint of each leg.

Since the proposed algorithm is to be implemented in a digital computer, it should be discretized. Notice that the majority of the variables in the proposed algorithm, such as $R_{df}\left(v\right)$, $\mu_{i}\left(v\right)$, $M_{i}\left(v\right)$, $T_{sw}\left(v\right)$, and so on, are all functions of the gait speed, except the inputs of the cyclic basis functions in Equation (24). Therefore, the overall algorithm can be implemented and simulated in real-time once g(t) is discretized into g(k), i.e.,

(25) $$g\left(k+1\right)=\left\{\begin{array}{cc} g\left(k\right)+\omega_{st}\left(v\right)T, & \mathrm{for}0\leq k<\frac{T_{sw}\left(v\right)}{T}\\ g\left(k\right)+\omega_{sw}\left(v\right)T, & \mathrm{for}\frac{T_{sw}\left(v\right)}{T}\leq k<\frac{T_{cycle}}{T} \end{array}\right.$$

where *T* is the sampling period. As g(t) is cyclic, g(k) is also cyclic, i.e.,

(26) $$g\left(k+n\frac{T_{cycle}}{T}\right)=g\left(k\right)$$

where *n* is any integer number.

## 5. Simulation Study

### 5.1. Simulation Environments

The proposed joint-angle trajectory generation algorithm for quadruped robots is verified by simulation studies in this section. In order to obtain reasonable information for verifying the proposed algorithm accurately in the simulation, the dynamic model of the simulation is necessary. In this simulation, Newtonian mechanics were applied to the dynamic model [33,34,35]. Newtonian mechanics describe the motion of rigid bodies. Additionally, Newtonian mechanics provide very accurate results as long as the model of a mechatronic system is an accurate interpretation of the actual physics. The detailed parameters of the Newtonian mechanics in this simulation are shown in Table 3.

Furthermore, indirect inverse dynamics based on a feedback control approach were used [36,37,38]. In the indirect inverse dynamics method, the input (i.e., the actuation force) is determined by the feedback controller such that an error between the desired motion by the proposed algorithm and the simulated output is small. In this simulation, the PD (proportional-derivative) feedback controller was applied as

(27) $$u=K_{P}\left(\theta_{d}-\theta_{m}\right)+K_{D}\left(\omega_{d}-\omega_{m}\right)$$

where *u* is the input, $\theta_{d}$ is the desired joint-angle trajectory by the proposed algorithm, $\theta_{m}$ is the simulated output by Newtonian mechanics model, and ω is the angular velocity. Furthermore, $K_{P}$ and $K_{D}$ were 15,000 and 2500, respectively, in this simulation. The simulation results were carried out using Matlab with the sampling period of 1 millisecond.

### 5.2. Simulation Results

**Figure 11:**
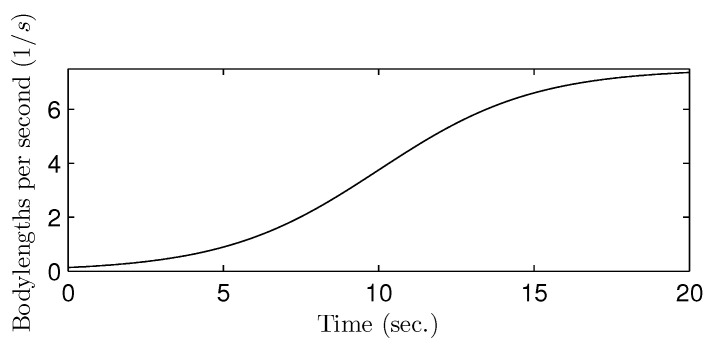
The speed graph with respect to the time used in the simulation.

The gait speed was set to smoothly increase from zero to 7.5 bodylength per second, which is 4.5 (m/s) for the bodylength of 0.6 (m), as shown in Figure 11.

**Figure 12:**
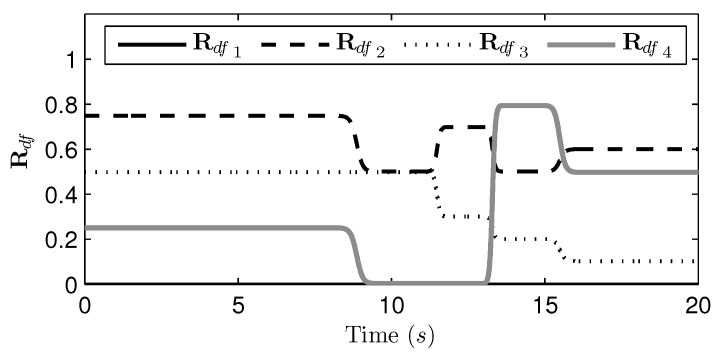
The four components of $R_{df}$ with respect to the time.

The first step of the proposed algorithm is to identify the likelihoods of gait phases for the current gait speed (i.e., $M_{i}\left(v\right)$) and to determine the desired ground contact sequences (i.e., $R_{df}\left(v\right)$). This process was introduced in Section 4.1. Figure 12 shows the four components of $R_{df}\left(v\right)$ with respect to the simulation time. Since the first component of $R_{df}\left(v\right)$ was used as the reference, it was zero for the entire time range, as shown in the figure. The remaining three components of $R_{df}\left(v\right)$ showed certain values with the desired intervals from the first component. It should be noted that the values of $R_{df}\left(v\right)$ were continuous and smooth for the entire time range, which implies that the smooth and continuous gait phase transition was achieved successfully.

**Figure 13:**
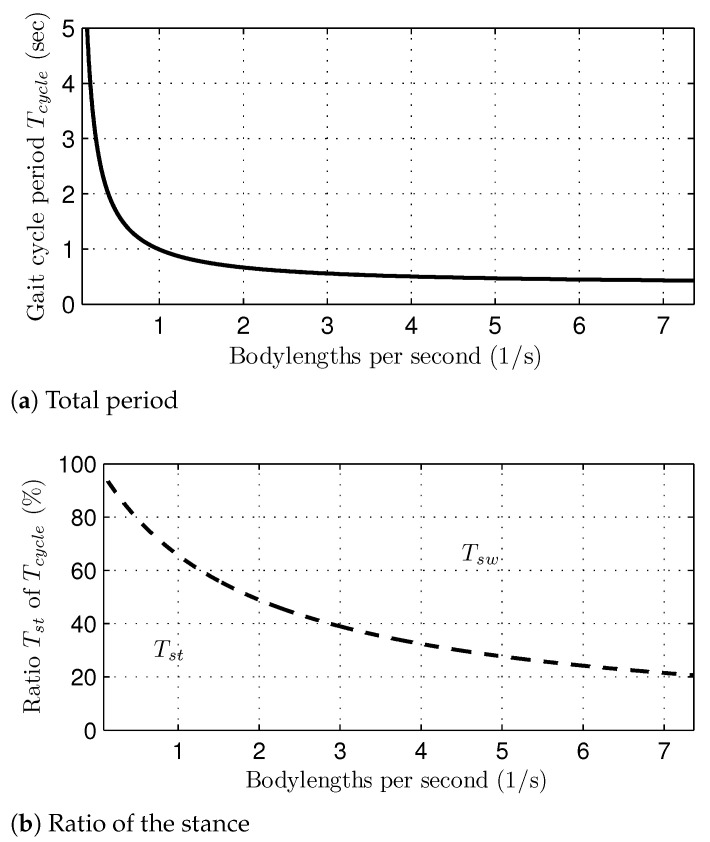
The total period and the stance ratio of one gait cycle in the simulation study: (**a**) total period and (**b**) ratio of the stance.

In addition to the identification of the likelihoods of gait phases and the desired ground contact sequences, the total period of one gait cycle is necessary for generating an input of cyclic basis functions, i.e., the index on the scaled gait cycle domain. Figure 13a shows the total period of one gait cycle calculated as in Equation (18). Notice that $T_{cycle}\left(v\right)$ was reduced as the gait speed increased, which implies that the cyclic locomotive motions occur more frequently at high speeds. In addition, a ratio between the swing and the stance periods was also changed according to the gait speed; the higher the gait speed, the lesser the ratio of the stance period, as shown in Figure 13b. Consequently, the ground contact time (i.e., the stance period) was shortened significantly as the gait speed increased, which may affect gait stability at high speeds.

**Figure 14:**
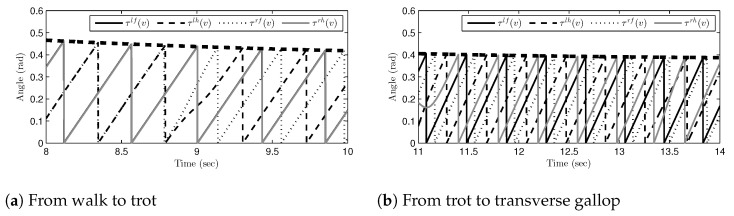
The desired time-intervals during gait phase transitions: (**a**) transitions from the walk phase to the trot phase and (**b**) transitions from the trot phase to the transverse gallop phase.

Since the total period of one gait cycle and the desired ground contact sequences were obtained, as shown in Figure 12 and Figure 13b, respectively, the desired time-intervals in the actual time domain can be calculated, as in Equation (17). Figure 14a,b show the desired time-intervals (i.e., $\tau^{lf}\left(v\right)$, …, $\tau^{rh}\left(v\right)$) calculated in the simulation. Notice that the time-intervals changed as the gait phases switched, and their magnitudes were reduced as the total period of one gait cycle was reduced. Nevertheless, the values of the time-intervals were smooth and continuous in the entire time range and successfully represented the desired ground contact sequences.

**Figure 15:**
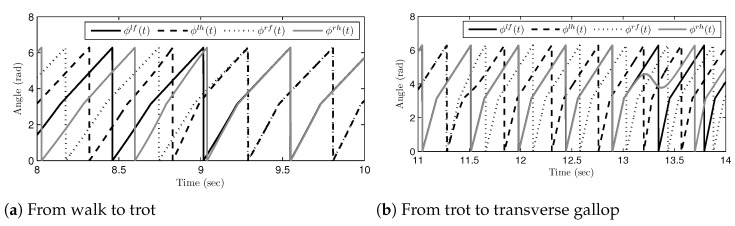
Indexes on the scaled gait cycle domain during gait phase transitions: (**a**) transitions from the walk phase to the trot phase and (**b**) transitions from the trot phase to the transverse gallop phase0.

Based on the calculated desired time-intervals, the scaled gait phase indexes can be obtained for the calculation of joint-angle trajectories, as shown in Figure 15. Remember that the scaled gait phase indexes are the input variables of the cyclic basis functions that represent the shapes of joint-angle trajectories. The scaled gait phase indexes continuously increased in the object’s continued locomotion; the values shown in Figure 15 are wrapped into the range of [0,2π) for better representation of the figure. The wrapping is automatically achieved in the proposed algorithm because g(t) in Equation (22) (or, g(k) in Equation (25)) is cyclic over [0,2π). The patterns shown in Figure 15a,b are different because the desired time-intervals and the rates of the indexes were different due to the gait speeds. The line shape of $\phi_{i}$ in one gait cycle is not linear, and the lines are bent in the middle of the gait cycle. It is because the kernel function makes Φ(k) from T(k). The kernel function composed of two linear functions that have different slopes: $\omega_{st}$ and $\omega_{sw}$. Thus, each $\phi_{i}$ has the same shape of the kernel function.

**Figure 16:**
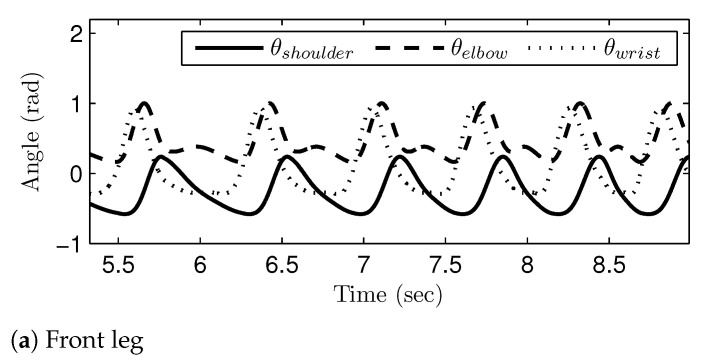

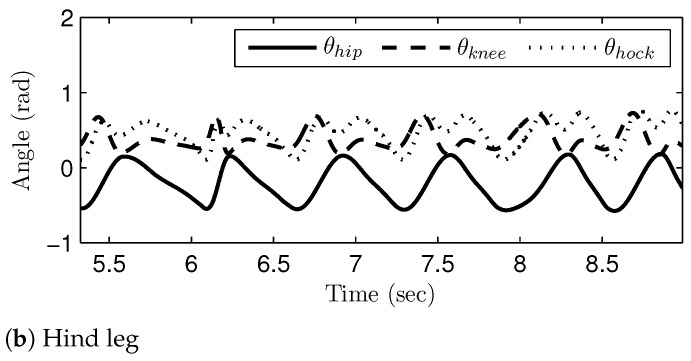
The join-angle trajectories when the gait phase is changed from the walk to canter: (**a**) front leg and (**b**) hind leg.

**Figure 17:**
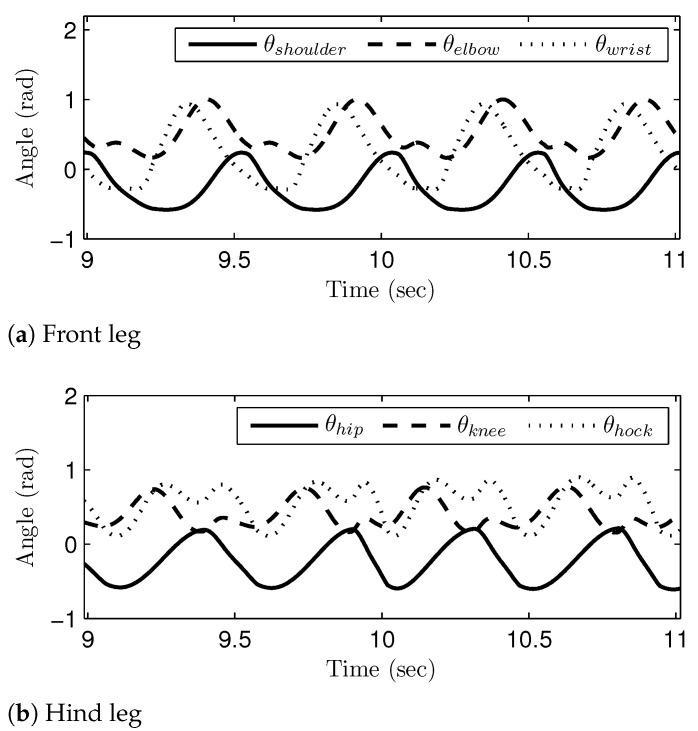
The join-angle trajectories when the gait phase is changed from the canter to transverse gallop: (**a**) front leg and (**b**) hind leg.

**Figure 18:**
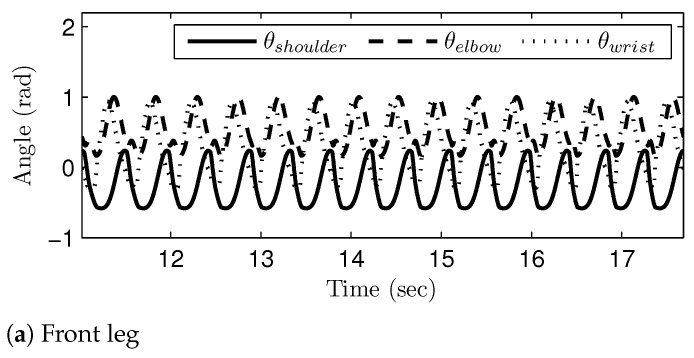

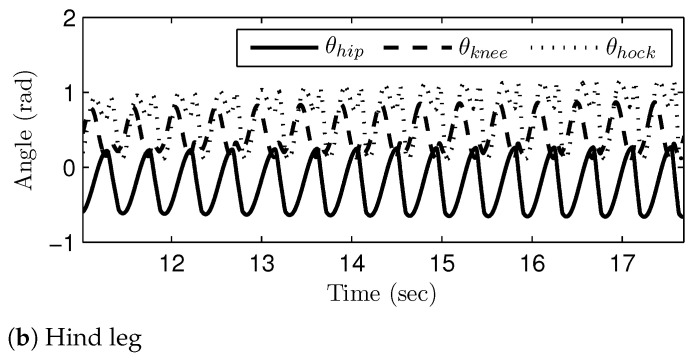
The join-angle trajectories when the gait phase is changed from the transverse gallop to rotatory gallop: (**a**) front leg and (**b**) hind leg.

Figure 16, Figure 17 and Figure 18 show the generated joint-angle trajectories in transitions of gait phases. It should be noted that the transition of the gait phases (i.e., the changes of the joint-angle amplitudes and intervals) took place smoothly in the generated joint-angle trajectories. The time axis in Figure 16 corresponds to the range of $v_{1E}$ and $v_{3S}$; remember that the gait speed was set to monotonically increase, as in Figure 11. Therefore, Figure 16 shows the gait phase transition from the walk phase to the canter phase, while passing through the trot phase. On the other hand, the time axis in Figure 17 corresponds to the range of $v_{3E}$ and $v_{4S}$, and thus, Figure 17 shows the gait phase transition from the canter phase to the transverse gallop phase. The time axis in Figure 18 shows the range of $v_{4E}$ and $v_{5S}$, which means that Figure 18 verifies the gait phase transition from the transverse gallop phase to the rotatory gallop phase. The sub-figures labeled as (a) and (b) represent the generated joint-angle trajectories for the front leg and the hind leg, respectively. The simulation results in Figure 16, Figure 17 and Figure 18 verify that the gait transition was realized smoothly and naturally without abrupt changes in the generated joint-angle trajectories.

**Figure 19:**
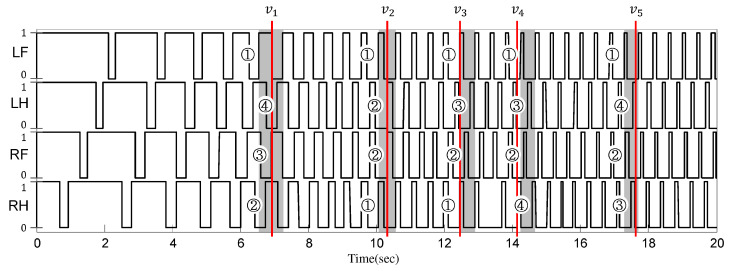
The ground contact indicators of four feet with respect to the time.

In order to observe the ground contact sequences in the simulation results, the stance and swing phases were analyzed, as shown in Figure 19. In the figure, the value of 1 means the stance phase, and the value of 0 represents the swing phase. Notice in the figure that the width of the stance phase was deceased as the gait speed increased, while that of the swing phase remained the same regardless of the gait speed. For each of the five characteristic speeds (i.e., $v_{1,2,\ldots ,5}$), one gait cycle is highlighted by gray boxes, as shown in Figure 19. In each gray box, the ground contact sequences can be observed as marked by numbers ①, ②, ③, and ④ in the figure. It should be noted that the numbers match the ground contact sequences shown in Table 2.

**Figure 20:**
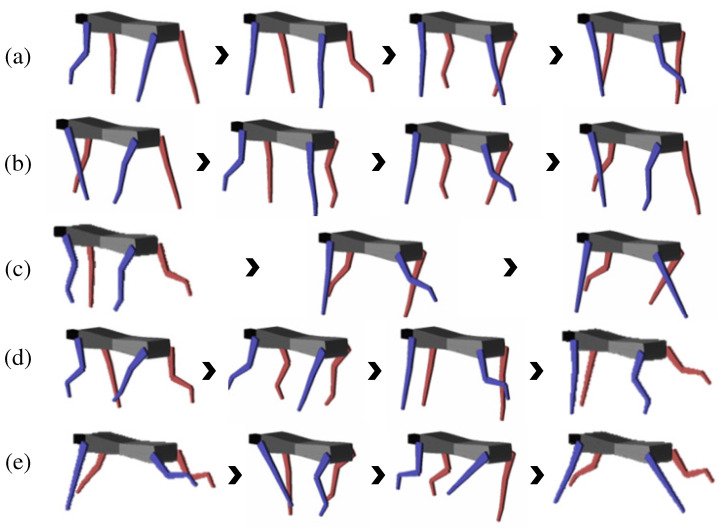
The leg motions of multiple gait phases from the proposed algorithm simulation: (**a**) the walk phase, (**b**) the trot phase, (**c**) the canter phase, (**d**) the transverse gallop phase, and (**e**) the rotatory gallop phase.

The generated joint-angle trajectories were applied to the three-dimensional model of a quadruped robot, as shown in Figure 20. Notice that the simulation model successfully realized locomotive motions in multiple gait phases. The proposed algorithm generated the joint-angle trajectories in real-time without changing any parameters in the simulation study.

## 6. Conclusions

In this paper, an algorithm that generates joint-angle trajectories for the control of a quadruped robot was proposed. The proposed algorithm was inspired by a quadruped animal and designed to realize locomotive motions by cyclic basis functions that represent the characteristic natures of the joint-angle trajectories of the animal. The proposed algorithm followed a hierarchical procedure: in the lowest level, it determined the most appropriate gait phase for a given gait speed. When the appropriate gait phase was not deterministic (e.g., the transition of gait phases), the fuzzy logic smoothly interpolated the likelihoods of two gait phases adjacent to the current gait speed. The estimated likelihoods of gait phases were utilized as weighting factors in the calculation of a resultant desired ground contact sequence. At the same time, the proposed method also calculated the stance and swing periods for the given speed, and the total period of one gait cycle was multiplied to the desired ground contact sequence in order to obtain the desired time-intervals in the actual time domain. At the middle level of the proposed algorithm, the indexes on the scaled gait cycle domain were calculated based on the desired gait phase (i.e., the desired time-intervals) and the gait speed. The indexes were the inputs of the cyclic basis functions. At the highest level, the joint-angle trajectories were calculated by the cyclic basis functions. The proposed algorithm was verified by the simulation results. It generated the joint-angle trajectories smoothly and continuously, even when the gait phases changed frequently. Since there was no abrupt change in the generated joint-angle trajectories, the proposed algorithm provided quadruped robots with effective reference joint-angle trajectories.

However, the proposed algorithm generates joint-angle trajectories considered in the sagittal plane only. In the real robot system, joint-angle trajectories have to be generated with considering three-dimensional space, including the frontal and transverse planes that are closely related to gait stability, balance, and locomotion performance (i.e., curve running, pass through the tilted and rough terrain). To generate the joint-angle trajectories in the three-dimensional space, the degree-of-freedoms should be added to the robot system, such as the abduction and adduction of the hip joint, rotation and flexion of the spine, movement of the pelvis and scapula, and the joint-angle trajectories of additional degree-of-freedoms should be obtained and formulated. Furthermore, the joint-angle trajectories of additional degree-of-freedoms will change according to the gait speed similarly to the degree-of-freedoms in the sagittal plane. Therefore, this paper has a meaningful contribution to the starting line of the research about obtaining and formulating the joint-angle trajectories of a robot system by taking account of the gait transition based on natural biology.

The feasibility of the proposed ideas was verified through the simulation results, but experimentation of the real robot system was not dealt with in this paper. The following research will introduce the proposed method’s experimental results with a high-speed quadruped robot. To progress the experiment of the high-speed quadruped robot, a feedback control method that robustly controls actuators to achieve the desired motions and guarantees the gait stability by reacting to disturbances has to be considered. In the real world, the more demanding conditions robots are exposed to, the more challenging the control of robotic legs becomes. As the gait speed increases, the frequency bandwidth of reference signals (i.e., desired motions) is also significantly enlarged. Thus, the significant enlargement of the closed-loop bandwidth of a control system is necessary. Consequently, high gain control with a high-speed data acquisition system is unavoidable, which also necessitates the accurate measurement of physical states without noise. Moreover, the dynamics of a robotic leg are highly uncertain and time-varying due to the ground contact and the ground condition, as well as the unknown payload. Therefore, a control algorithm that can achieve both great control performance and stability robustness is essential for the effective control of a quadruped robot.

Furthermore, an external disturbance (e.x., ground reaction force, impact, change of the load condition. etc.) has to be considered in terms of the control method. A disturbance observer (DOB) is a good choice for dealing with external disturbance. The DOB estimates external disturbances by comparing an actual measurement with a simulated output. By filtering the output discrepancy with an inverse of the system model, the estimation of the exogenous disturbance is achieved. The DOB can also be applied as a feedback controller, in which case the disturbance is rejected, and the overall system is controlled to follow the nominal model. Since it attenuates the model variation and makes the closed-loop system robust to external disturbances, the DOB is appropriate for the control of the quadruped robot.

## Figures and Tables

**Table 1:** Speed, percent of stance period, stance time, and swing time during one gait cycle.

Phase	Walk Phase		Trot Phase		Gallop Phase	
**Gait Period**	**Front**	**Hind**	**Front**	**Hind**	**Front**	**Hind**
Speed, bodylengths per second, (1/s)	1.98		4.48		7.23	
(meters per second, (m/s))	(1.19)		(2.69)		(4.34)	
Average stance period (s)	0.55	0.48	0.23	0.21	0.14	0.16
(standard deviation)	0.01	0.02	0.06	0.04	0.07	0.07
Average Swing period (s)	0.32	0.37	0.34	0.34	0.31	0.32
(standard deviation)	0.02	0.02	0.05	0.04	0.08	0.09
Average ratio of stance period, *d*	0.63	0.56	0.41	0.39	0.31	0.34

**Table 2:** Desired time-intervals, $R_{di}$, according to gait phases.

Phase	Walk	Trot	Canter	T.Gallop	R.Gallop
Notation	$R_{d1}$	$R_{d2}$	$R_{d3}$	$R_{d4}$	$R_{d5}$
Values	$\left[\begin{array}{c} 0\\ 0.75\\ 0.5\\ 0.25 \end{array}\right]$	$\left[\begin{array}{c} 0\\ 0.5\\ 0.5\\ 0 \end{array}\right]$	$\left[\begin{array}{c} 0\\ 0.7\\ 0.3\\ 0 \end{array}\right]$	$\left[\begin{array}{c} 0\\ 0.5\\ 0.2\\ 0.8 \end{array}\right]$	$\left[\begin{array}{c} 0\\ 0.6\\ 0.1\\ 0.5 \end{array}\right]$

**Table 3:** Parameters of the simulation.

Segment	Length (m)	Weight (kg)	Moment of Inertia (kgm${}^{2}$)
Head	0.2	1	0.3
Upper trunk	0.35	5	0.5
Lower trunk	0.35	2	0.5
Front upper link	0.2	0.5	0.3
Front middle link	0.2	0.3	0.2
Front lower link	0.1	0.2	0.1
Hind upper link	0.2	3	0.3
Hind middle link	0.2	2	0.2
Hind lower link	0.1	0.5	0.1

## Data Availability

Not applicable.

## References

[1] Holmes P., Full R.J., Koditschek D., Guckenheimer J. (2006). The Dynamics of Legged Locomotion: Models, Analyses, and Challenges. Soc. Ind. Appl. Math..

[2] Li Y., Li B., Ruan J., Rong X. Research of Mammal Bionic Quadruped Robots: A Review. Proceedings of the 2011 IEEE 5th International Conference on Robotics, Automation and Mechatronics.

[3] Raibert M., Blankespoor K., Nelson G., Playter R., BigDog Team BigDog, the Rough-Terrain Quadruped Robot. Proceedings of the 17th World Congress The International Federation of Automatic Control.

[4] Boston Dynamics BigDog Overview (Updated March 2010). https://www.youtube.com/watch?v=cNZPRsrwumQ.

[5] Boston Dynamics LS3-Legged Squad Support System. https://www.youtube.com/watch?v=R7ezXBEBE6U.

[6] Boston Dynamics Spot Autonomous Navigation. https://www.youtube.com/watch?v=Ve9kWX_KXus.

[7] Boston Dynamics Spot’s Got an Arm. https://www.youtube.com/watch?v=6Zbhvaac68Y.

[8] Boston Dynamics Spot on Site: Construction Solution. https://www.youtube.com/watch?v=0NYJ_9FIHZA.

[9] Valenzuela A.K., Kim S. Optimally Scaled Hip-Force Planning: A Control Approach for Quadrupedal Running. Proceedings of the 2012 IEEE International Conference on Robotics and Automation.

[10] Bledt G., Powell M.J., Katz B., Di Carlo J., Wensing P.M., Kim S. MIT Cheetah 3: Design and Control of a Robust, Dynamic Quadruped Robot. Proceedings of the 2018 IEEE International Conference on Intelligent Robots and Systems.

[11] Biomimetics MIT Dynamic Locomotion in the MIT Cheetah 3 through Convex Model Predictive Control. https://www.youtube.com/watch?v=q6zxCvCxhic.

[12] Katz B., Carlo J.D., Kim S. Mini Cheetah: A Platform for Pushing the Limits of Dynamic Quadruped Control. Proceedings of the 2019 International Conference on Robotics and Automation.

[13] Massachusetts Institute of Technology Backflipping MIT Mini Cheetah. https://www.youtube.com/watch?v=xNeZWP5Mx9s.

[14] Kim T., Park S., Yi B.J. A Composite Algorithm for Flow Rate Reduction and Stable Body Trajectory Generation in a Hydraulic Actuated Quadruped Robot with Kinematic Redundancy. Proceedings of the 2011 IEEE International Conference on Mechatronics and Automation.

[15] Liu C., Chen Y., Zhang J., Chen Q. CPG Driven Locomotion Control of Quadruped Robot. Proceedings of the 2009 IEEE International Conference on Systems, Man, and Cybernetics.

[16] Crespi A., Ijspeert A.J. (2008). Online Optimization of Swimming and Crawling in an Amphibious Snake Robot. IEEE Transection Robot..

[17] Liu A., Zhang X.Y., Zhang K.G. (2013). Gait Transition of Quadruped Robot Using Time Sequence Control Based on Finite-State Machine. Appl. Mech. Mater..

[18] Hussain R., Zielinska T., Hexel R. (2019). Finite State Automaton based Control System for Walking Machines. Int. J. Adv. Robot. Syst..

[19] Ding Y., Pandala A., Li C., Shin Y.H., Park H.W. (2021). Representation-Free Model Predictive Control for Dynamic Motions in Quadrupeds. IEEE Transection Robot..

[20] Taylor C.R. (1978). Why Change Gaits? Recruitment of Muscles and Muscle Fibers as a Function of Speed and Gait. Integr. Comp. Biol..

[21] Biewener A.A. (2006). Patterns of Mechanical Energy Change in Tetrapod Gait: Pendula, Springs and Work. J. Exp. Zool..

[22] ITO S., Sahashi Y., Sasaki M. Learning Scheme of Multiple-patterns in Quadruped Locomotion Using CPG Model. Proceedings of the 2008 Society of Instrument and Control Engineer Annual Conference.

[23] Boston Dynamics Introducing Spot Classic (Previously Spot). https://www.youtube.com/watch?v=M8YjvHYbZ9w.

[24] Li J., Wang J., Yang S.X., Zhou K., Tang H. (2016). Theory and Applications of Bioinspired Neural Intelligence for Robotics and Control. Comput. Intell. Neurosci..

[25] Chen J., San H., Wu X. (2019). Gait Regulation of a Bionic Quadruped Robot with Antiparallelogram Leg Based on CPG Oscillator. Complexity.

[26] Alexander R.M. (1984). The Gait of Bipedal and Quadrupedal Animals. Int. J. Robot. Res..

[27] McMahon T.A. (1985). The Role of Compliance in Mammalial Running Gaits. J. Exp. Biol..

[28] Debrand M.H. (1961). Further Studies on Locomotion of the Cheetah. J. Exp. Biol..

[29] Fastec Co. http://www.fastecimaging.com/products/high-speed-cameras/handheld-cameras/ts3-100-s.

[30] Mamdani E.H. (1977). Application of Fuzzy Logic to Approximate Reasoning Using Linguistic Synthesis. IEEE Trans. Comput..

[31] Iancu I., Dadios F. (2012). A Mamdani Type Fuzzy Logic Controller. Fuzzy Logic-Controls, Concepts, Theories and Applications.

[32] Izquierdoa S.S., Izquierdo L.R. (2018). Mamdani Fuzzy Systems for Modelling and Simulation: A Critical Assessment. J. Artif. Soc. Soc. Simul..

[33] My C.A., Bien D.X. (2020). New Development of the Dynamic Modeling and the Inverse Dynamic Analysis for Flexible Robot. Int. J. Adv. Robot. Syst..

[34] Faber H., van Soest A.J., Kistemaker D.A. (2018). Inverse Dynamics of Mechanical Multibody Systems: An Improved Algorithm that Ensures Consistency between Kinematics and External Forces. PLoS ONE.

[35] Otten E. (2003). Inverse and Forward Dynamics: Models of Multi-body Systems. Philos. Trans. R. Soc. Biol. Sci..

[36] Sharifzadeha M., Ariana A., Salimia A., Masoulehc M.T., Kalhor A. (2018). An Experimental Dynamic Identification and Control of an Overconstrained 3-DOF Parallel Mechanism in Presence of Variable Friction and Feedback Delay. Robot. Auton. Syst..

[37] Kappler D., Meier F., Ratliff N., Schaal S. A New Data Source for Inverse Dynamics Learning. Proceedings of the 2017 IEEE/RSJ International Conference on Intelligent Robots and Systems.

[38] Murray-Smith D.J. (2011). Feedback Methods for Inverse Simulation of Dynamic Models for Engineering Systems Applications. Math. Comput. Model. Dyn. Syst..

